# Potential Relationship Between YTHDF3 and CFTR in Myocardial Ischemia–Reperfusion Injury

**DOI:** 10.1111/jcmm.71240

**Published:** 2026-06-09

**Authors:** Baoxin Tang, Chenying Zhu, Heqing Wang, Mingkui Gao, Tieyan Li

**Affiliations:** ^1^ Department of Cardiology Shanghai East Hospital, Tongji University School of Medicine Shanghai China; ^2^ Department of Heart Failure Shanghai East Hospital, Tongji University School of Medicine Shanghai China; ^3^ Department of Cardiovascular Surgery Shanghai East Hospital, Tongji University School of Medicine Shanghai China

**Keywords:** AMPK pathway, CFTR, myocardial ischemia–reperfusion injury, YTHDF3

## Abstract

Myocardial ischemia–reperfusion injury (MIRI) is a common pathophysiological process in reperfusion therapy following myocardial infarction. It exacerbates myocardial damage and negatively impacts patient prognosis. This study aims to explore potential regulatory targets and related mechanisms in MIRI. Using the GSE123342 dataset, differentially expressed genes (DEGs) related to myocardial infarction‐associated conditions were screened and a protein–protein interaction network was constructed. Core genes were identified through weighted gene co‐expression network analysis (WGCNA), followed by GO and KEGG enrichment analyses. The GSE6381 dataset was used to examine core gene expression, and ROC curve analysis was performed as an exploratory assessment of group separation. A transcription factor regulatory network for CFTR was constructed using the KnockTF database. An oxygen–glucose deprivation/reoxygenation (OGD/OGR) model was established in AC16 cells, and plasmid overexpression of YTHDF3 and CFTR was used to examine their effects on OGD/OGR‐induced cell injury. The interaction between YTHDF3 and CFTR mRNA was examined by RIP‐qPCR experiments, and CFTR mRNA stability was assessed using an actinomycin D assay. Additionally, the effects of YTHDF3 and CFTR overexpression were examined in a rat I/R model. We found in the GSE123342 dataset, 5444 differentially expressed mRNAs were identified, with CFTR significantly upregulated. Correlation analysis revealed 6651 genes related to CFTR. WGCNA identified 120 hub genes, with the MEyellow module significantly associated with MIRI. These genes were mainly enriched in ABC transporters and the AMPK pathway. In the GSE6381 dataset, CFTR and YTHDF3 showed differential expression and ROC analysis showed preliminary group separation. CFTR was closely related to multiple transcription factors, including TP53, STAT3 and TFAP4. In AC16 cells, OGD/OGR decreased YTHDF3 expression and increased CFTR expression. YTHDF3 overexpression reduced CFTR expression and aggravated OGD/OGR‐induced injury, as shown by reduced cell viability and proliferation and increased apoptosis. Additional CFTR overexpression partly attenuated these changes. In the rat I/R model, YTHDF3 overexpression increased myocardial infarct size and impaired cardiac function, whereas CFTR overexpression attenuated the injury associated with YTHDF3 overexpression. CFTR knockdown aggravated OGD/OGR‐induced cellular injury and was associated with changes in AMPK signalling. Together, these findings suggest that YTHDF3 may be associated with MIRI and CFTR‐related changes. Changes in AMPK signalling were also observed, but the underlying mechanism still needs further validation. These results provide preliminary evidence for a possible relationship between YTHDF3 and CFTR in MIRI.

**Abbreviations**
ANOVA: analysis of variance
AUC: area under curve
BP: biological process
CC: cellular component
CCK‐8: Cell Counting Kit 8
CK: cardiac‐type creatine kinase
CK‐MB: creatine kinase isoenzyme MB
DEGs: differentially expressed genes
DO: disease ontology
ECG: electrocardiogram
FBS: fetal bovine serum
FC: fold change
GEO: gene expression omnibus
GO: Gene Ontology
GSEA: Gene Set Enrichment Analysis
HE: haematoxylin and eosin
I/R: ischemia–reperfusion
KEGG: Kyoto Encyclopedia of Genes and Genomes
LDH: lactate dehydrogenase
LVEDd: left ventricular end‐diastolic diameter
LVEDs: end‐systolic diameter
LVEF: ejection fraction
LVFS: fractional shortening
ME: module eigengene
MF: molecular function
MIRI: myocardial ischemia–reperfusion injury
OGD/OGR: oxygen‐glucose deprivation/reoxygenation
RIP‐qPCR: RNA immunoprecipitation
ROC: receiver operating characteristic
RT‐qPCR: real‐time quantitative polymerase chain reaction
SD: Sprague–Dawley
SD: standard deviation
TOM: Topological Overlap Matrix
TTC: 2,3,5‐Triphenyltetrazolium Chloride
WGCNA: weighted gene co‐expression network analysis
α‐HBDH: α‐hydroxybutyrate dehydrogenase

## 1 Introduction

Myocardial ischemia–reperfusion injury (MIRI) is a frequent pathophysiological process in patients with acute myocardial infarction following reperfusion therapy [1]. It is characterized by the exacerbation of myocardial injury after the restoration of blood flow to ischemic myocardium, which can induce arrhythmias, cardiac dysfunction and even heart failure, thereby severely affecting patient prognosis [2]. In recent years, with the widespread application of reperfusion techniques such as thrombolysis and interventional therapy, the clinical attention to MIRI has significantly increased. Nevertheless, specific therapeutic approaches for MIRI are still lacking [3]. The pathogenesis of MIRI is complex and involves multiple mechanisms, including oxidative stress, apoptosis, inflammatory responses and calcium overload and it is also closely related to several signalling pathways. For example, abnormal activation or inhibition of AMPK and cAMP signalling pathways can influence the extent of myocardial injury by regulating metabolic balance and cell survival [4, 5, 6]. However, the molecular regulatory network underlying MIRI has not yet been fully elucidated, and there is a scarcity of targeted interventions in clinical practice. Thus, further investigation into the core mechanisms of MIRI is essential.

In recent years, studies have found that RNA epigenetic modifications (such as m6A methylation) have emerged as a possible mechanism for regulating MIRI by affecting mRNA stability, translation efficiency and subcellular localization [7]. m6A regulators are proposed key molecules involved in RNA methylation modifications, including methyltransferases, demethylases and readers (such as YTHDF family proteins) [8]. By recognizing m6A modification sites on RNA, these regulators can influence processes like mRNA stability and translation efficiency, thus playing a role in gene expression regulation [9, 10]. In MIRI, dynamic changes in m6A modification may affect myocardial cell survival, proliferation and apoptosis by regulating the expression of key genes. For instance, m6A readers may regulate the expression of stress response‐related genes during ischemia–reperfusion by recognizing modification sites on target gene RNA, thereby influencing the degree of myocardial injury [11, 12]. However, the specific roles of particular m6A regulators and the target gene regulation mechanisms in MIRI still require further investigation.

Bioinformatics technology, with its advantages in efficiently integrating multi‐omics data and mining key molecules and regulatory networks, has become an important tool for disease mechanism research [13]. Using methods such as high‐throughput data analysis (e.g., mining Gene Expression Omnibus (GEO) datasets), screening differentially expressed genes (DEGs) and performing weighted gene co‐expression network analysis (WGCNA), bioinformatics can quickly identify core genes and potential regulatory factors related to diseases, greatly enhancing research efficiency [14]. In the study of MIRI, bioinformatics has been used to screen DEGs, construct gene interaction networks and predict signalling pathways, providing direction for subsequent mechanism validation [15, 16]. In the preliminary stage of this study, bioinformatics analysis was utilized to screen the highly expressed CFTR gene from transcriptomic data related to MIRI and further explore its potential m6A regulators, laying the foundation for investigating their roles in MIRI. Given the abnormal expression of CFTR in MIRI and the uncertainty of its functional role, this study aimed to examine the possible relationship between YTHDF3 and CFTR expression and to explore their relevance to MIRI.

## 2 Materials and Methods

### 2.1 Acquisition of Disease Targets

Transcriptomic microarray data related to ‘Myocardial Ischemia Reperfusion’ were obtained from the GEO database (https://www.ncbi.nlm.nih.gov/geo/). The datasets were searched using ‘Myocardial Ischemia Reperfusion’ as the keyword, and datasets containing human samples were retained. The GSE123342 dataset included samples from acute myocardial infarction (65 cases), 30 days post‐infarction (64 cases), 1‐year post‐infarction (37 cases), stable coronary heart disease (22 cases) and 4 technical replicate samples. This dataset was selected because it contains different myocardial infarction‐related stages and has a relatively large sample size. For differential expression analysis, myocardial infarction‐related samples were treated as the disease‐related group, and stable coronary heart disease samples were used as the comparison group. Technical replicate samples were not treated as independent biological samples. The Bioconductor package in R was used for background correction, normalization and expression value calculation. Differential expression analysis was performed using the limma package. Genes with adjusted *p* value < 0.05 and fold change ≥ 1.50‐fold (|log2FC| ≥ 0.58) were considered differentially expressed genes. The adjusted *p* value was used to account for multiple testing. Heatmaps and volcano plots were generated in R. For volcano plots, adjusted *p* values were transformed into −log10 values, and genes were classified as upregulated, downregulated, or non‐significant according to log2FC and adjusted *p* value. The DEGs were ranked by log2FC, and Gene Set Enrichment Analysis (GSEA) was then performed. MIRI‐related targets were obtained from GeneCards (https://www.genecards.org/) and OMIM (https://omim.org/). The target lists were merged, and duplicate genes were removed. Gene names were checked using the UniProt database. MIRI‐related targets were imported into Metascape (https://metascape.org/) for PPI network construction. The m6A regulator list was obtained from the GSEA database. GeneCards and OMIM were used because they provide complementary information on disease‐related genes, while the m6A gene list was used to focus the screening on RNA methylation‐related regulators. The intersection strategy was used to prioritize genes related to MIRI‐associated DEGs, CFTR‐related genes, WGCNA hub genes and m6A regulation. This step was used for candidate‐gene prioritization and was not interpreted as direct evidence of a mechanistic relationship. The main databases, software tools, R packages and filtering thresholds used in this workflow are described above to improve reproducibility.

### 2.2 Clustering of WGCNA

To reduce noise from low‐variance genes, genes in the highest quartile of variance were selected for WGCNA, as these genes were more likely to reflect meaningful biological variation. These genes were used in the R “WGCNA” package to create a WGCNA for MIRI. A sample clustering tree was generated to detect obvious outlier samples before network construction. The optimal soft threshold β was determined via scale‐free network analysis. The soft‐thresholding power was set to *β* = 27 when the scale‐free topology fit index reached an acceptable level. The optimal β was selected to maximize the network's scale‐free topology, which is essential for obtaining reliable gene co‐expression modules. The adjacency matrix was computed using β power, and a Topological Overlap Matrix (TOM) was established to assess gene similarity and construct a hierarchical clustering tree. Modules were delineated and merged using the dynamic hybrid cutting method, and a gene dendrogram was created. The main WGCNA parameters were set as follows: networkType = signed, minModuleSize = 30 and mergeCutHeight = 0.25. The module eigengene (ME) for each module was calculated, and the correlation between ME and MIRI clinical traits was determined using Pearson's correlation coefficient. Modules with |correlation coefficient| ≥ 0.60 and *p* < 0.05 were considered MIRI‐related modules. This threshold was used to focus on modules showing a moderate‐to‐strong association with the disease‐related trait. The module most strongly associated with MIRI was identified as the key module, and further screening of genes within this module was performed. Genes in the key module were further screened according to module membership and gene significance, with module membership > 0.80 and gene significance > 0.20 used as the criteria for candidate hub genes. These criteria were used to prioritize genes closely related to both the key module and the disease‐related trait. The interactions between hub genes related to MIRI were examined. The intersection of MIRI‐related DEGs and MIRI‐related hub genes was identified using R software (https://www.r‐project.org/) and a Perl script. The results were then input into Venny 2.1 software (http://bioinfogp.cnb.csic.es/tools/venny/index.html) to generate a Venn diagram. For subsequent correlation screening, both Pearson and Spearman correlation analyses were performed and genes with *p* < 0.05 in the correlation analysis were retained. Both methods were used to reduce dependence on a single correlation approach.

### 2.3 Gene Ontology (GO) and Kyoto Encyclopedia of Genes and Genomes (KEGG) Enrichment Analysis

R package ‘clusterProfilerGO.R’ and the Perl language were employed to conduct GO analysis on the core targets. GO analysis was mainly used to describe the functions of gene products in three areas: Cellular Component (CC), Molecular Function (MF) and Biological Process (BP). Furthermore, KEGG pathway enrichment analysis was performed using the ‘clusterProfilerKEGG.R’ package, and relevant signalling pathway diagrams were drawn with the ‘pathview’ package. Core pathway enrichment levels were assessed via enrichment factor values, providing insights into the biological functions and signalling mechanisms related to MIRI core genes.

### 2.4 Relative Expression of Core Targets and Receiver Operating Characteristic (ROC) Assessment

MIRI‐related expression matrix data were obtained from the GEO database for external validation. These data include transcriptomic expression matrix files for MIRI. The ggpubr package was used to analyse the relative expression levels of the core targets identified in MIRI data and to generate box plots. The external validation data were selected for their comparability with the internal dataset, ensuring that both datasets reflect similar biological conditions. Normalized MIRI and core target expression data were integrated with clinical data. ROC curves for the core genes were constructed using the survivalROC package in R, with related data processing performed using survival, caret, glmnet and survminer. The area under the ROC curve (AUC) was calculated as an exploratory measure of group separation in the analysed datasets.

### 2.5 Immune Infiltration and Immune Function Analysis of MIRI‐Related Core Targets

The CIBERSORT package was employed to perform deconvolution analysis on the obtained MIRI expression matrix data. Using standardized gene expression data, we inferred the relative proportions of different immune cell types in MIRI tissue samples, offering a detailed profile of immune cell infiltration. This process generated an expression matrix for MIRI‐related infiltrating immune cells, which was screened using a Perl script. The limma package in R was used for background correction, normalization and expression value calculation of the microarray data. CIBERSORT was then used to estimate and analyse the immune cell composition in MIRI samples. Because of the limited sample size of some validation datasets, the immune infiltration analysis was considered exploratory and was not used as independent validation of the main findings.

### 2.6 Construction of Core Gene and CFTR‐Transcription Factor Regulatory Network and Enrichment Analysis

Transcription factors related to core m6A genes and the CFTR gene were identified using the KnockTF database (http://www.licpathway.net/KnockTF/index.php). The UniProt database (https://www.uniprot.org/) was used to match and correct target gene names. The PPI network of CFTR and its transcription factors was then constructed using Cytoscape 3.7.2 software (https://cytoscape.org/). In this analysis, we focused on identifying transcription factors with known roles in m6A regulation, as they are likely to influence CFTR gene expression. Additionally, Disease Ontology (DO), GO and KEGG enrichment analyses were conducted on potential target sites of CFTR's transcription factors to provide insights into the biological relevance of these interactions. Pathway annotation was conducted based on the KEGG database [17].

### 2.7 Cell Culture

Human immortalized cardiomyocyte AC16 cells (CTCC‐003‐0014, MeisenCTCC) were cultured in DMEM/F12 medium (AC16‐CM, MeisenCTCC) supplemented with 10% fetal bovine serum (FBS) and 1% Penicillin/Streptomycin. The cells were incubated at 37°C in a 5% CO_2_ incubator and grown to the logarithmic phase (80%–90% confluence). Once the cells reached the required confluence, they were used for the subsequent experiments.

### 2.8 Oxygen–Glucose Deprivation/Reoxygenation (OGD/OGR) Model Cell Model Construction

To establish the OGD/OGR model, the culture medium was replaced with glucose‐free and serum‐free DMEM, and the cells were incubated in a hypoxic environment consisting of 94% N_2_, 5% CO_2_ and 1% O_2_ for 3 h at 37°C. This hypoxic treatment simulates ischemic conditions. Following the hypoxia treatment, the cells were immediately transferred back to complete DMEM/F12 medium containing 10% FBS and placed in a regular incubator under normal conditions (37°C, 5% CO_2_, 95% air) for 6 h to allow for reoxygenation.

### 2.9 Plasmid Construction

The pECMV‐3XFLAG‐YTHDF3 plasmid (P4927) and pCDNA3.1‐CFTR (human)‐3XFLAG (P27726) were synthesized by Wuhan Miaoling Biotechnology Co. Ltd.

The core vector backbone used for overexpression was pCDNA3.1(+). The human CFTR coding sequence (NM_000492.4) was retrieved from the gene database. PCR primers were designed to amplify the open reading frame (ORF) of CFTR, with HindIII and XhoI restriction sites added to the upstream and downstream primers, respectively. High‐fidelity PCR was performed to amplify the CFTR cDNA fragment, which was then purified by gel extraction. For cloning, the pCDNA3.1(+) vector and the PCR product were both digested with HindIII and XhoI restriction enzymes. The linearized vector and the insert were then purified. The digested vector and insert were ligated using T4 DNA ligase and transformed into competent *E. coli* cells (TOP10), which were plated on LB agar plates containing ampicillin for overnight incubation. Colony PCR was performed to screen for positive clones, and plasmid DNA was extracted from the positive clones. The clones were then subjected to restriction enzyme digestion and sequencing to confirm the correct insertion of CFTR without mutations and in the correct orientation.

### 2.10 Cell Transfection

For overexpression experiments, AC16 cells were transfected with the pECMV‐3XFLAG‐YTHDF3 or pCDNA3.1‐CFTR (human)‐3XFLAG plasmids using Lipofectamine 2000 (11668‐027, Invitrogen) according to the manufacturer's protocol. Before transfection, AC16 cells were seeded in 6‐well plates at 1 × 10^5^ cells per well and cultured until they reached approximately 70%–80% confluence. For each well, 2 μg plasmid and 5 μL Lipofectamine 2000 were separately diluted in serum‐free medium, mixed gently and incubated for 15–20 min at room temperature before being added to the cells. The cells were incubated for 48 h following transfection and then subjected to OGD/OGR treatment or other experimental conditions. The overexpression efficiency of YTHDF3 and CFTR was confirmed by RT‐qPCR and Western blot before subsequent experiments. For siRNA knockdown, the cells were transfected with siRNAs targeting YTHDF3 (siYTHDF3_1, siYTHDF3_2, siYTHDF3_3) or CFTR (siCFTR_1, siCFTR_2, siCFTR_3) using the same Lipofectamine 2000 protocol. The final concentration of siRNA was 50 nM. The cells were incubated for 48 h post‐transfection before being used in subsequent experiments. Knockdown efficiency was assessed by RT‐qPCR, and the reduction efficiency was above 85%. The sequences of target‐specific siRNAs are provided in Table S1. A nonspecific negative control siRNA from the same supplier was used as the NC control.

### 2.11 Cell Groups for Experimentation

The experiments were divided into two groups: Control group (AC16 cells were cultured under normal conditions without special treatment) and OGD/OGR group. The overexpression plasmid of YTHDF3 was constructed, and the groups were divided as follows: NC (AC16 cells transfected with the control plasmid) and oeYTHDF3 (AC16 cells transfected with the YTHDF3 overexpression plasmid). Further grouping was as follows: NC (AC16 cells transfected with the control plasmid), oeYTHDF3 (AC16 cells transfected with the YTHDF3 overexpression plasmid), NC+OGD/OGR (after transfecting AC16 cells with the control plasmid, the OGD/OGR model was established) and oeYTHDF3+OGD/OGR (after transfecting AC16 cells with the YTHDF3 overexpression plasmid, the OGD/OGR model was established). In addition, CFTR was overexpressed, and the groups were divided as follows: NC (AC16 cells transfected with the control plasmid), NC+OGD/OGR (after transfecting AC16 cells with the control plasmid, the OGD/OGR model was established), oeYTHDF3+OGD/OGR (after transfecting AC16 cells with the YTHDF3 overexpression plasmid, the OGD/OGR model was established) and oeYTHDF3+oeCFTR + OGD/OGR (after co‐transfecting AC16 cells with the YTHDF3 and CFTR overexpression plasmids, the OGD/OGR model was established). The same amount of empty vector was used in the corresponding control groups to keep the total plasmid amount consistent. For CFTR knockdown experiments, AC16 cells were divided into the following groups: NC (cells transfected with negative control siRNA), si‐CFTR_1, si‐CFTR_2 and si‐CFTR_3, representing three different CFTR siRNAs used for knockdown. In the OGD/OGR treatment experiments, the cells were also divided into NC (Control group), NC+OGD/OGR, si‐CFTR+OGD/OGR and si‐CFTR+AMPK‐IN‐3+OGD/OGR, with AMPK inhibition added alongside CFTR knockdown and OGD/OGR treatment. AMPK‐IN‐3 was used at 50 μM for 48 h. AMPK‐IN‐3 was dissolved in DMSO, and the same volume of DMSO was added to the corresponding control cells. For YTHDF3 knockdown, the groups included: NC (cells transfected with negative control siRNA), si‐YTHDF3_1, si‐YTHDF3_2 and si‐YTHDF3_3, each using a different siRNA to target YTHDF3. In the YTHDF3 knockdown and OGD/OGR treatment experiments, the groups were: NC, si‐YTHDF3, NC+OGD/OGR, and si‐YTHDF3+OGD/OGR. Finally, for combined YTHDF3 and CFTR knockdown with OGD/OGR, the groups included: NC, NC+OGD/OGR, si‐YTHDF3+OGD/OGR and si‐YTHDF3+si‐CFTR+OGD/OGR. For all knockdown experiments, negative control siRNA was used as the NC control.

### 2.12 Real‐Time Quantitative Polymerase Chain Reaction (RT‐qPCR)

CFTR and YTHDF3 levels in AC16 cells were evaluated using RT‐qPCR technology. The initial step of the experiment was to obtain total RNA using the TriQuick Reagent Total RNA Extraction Kit (R1100, Solarbio), followed by transcribing RNA into cDNA with 5 × RT SuperMix for qPCR (K1074, APEXbio). Quantitative detection of the target genes was performed on an iQ5 Multicolor Real‐Time PCR Detection System (582BR 005500, BIO‐RAD) using 2 × SYBR Green qPCR Master Mix (K1070, APEXbio). GAPDH was used as the reference gene in the experiment, and relative quantification was carried out using the 2^−ΔΔCt^ method. The detailed information on the primer sequences is shown in Table 1.

**TABLE 1:** The primers used in this study.

Names	Sequences (5′ → 3′)	Lengths (bp)
H‐CFTR‐F1	AAAAGGCCAGCGTTGTCTCC	211
H‐CFTR‐R1	AAACATCGCCGAAGGGCATTA	
H‐YTHDF3‐F1	TCAGAGTAACAGCTATCCACCA	136
H‐YTHDF3‐R1	GGTTGTCAGATATGGCATAGGCT	
H‐GAPDH‐F1	CATCATCCCTGCCTCTACTGG	259
H‐GAPDH‐R1	GTGGGTGTCGCTGTTGAAGTC	

### 2.13 Western Blot

Total protein was extracted using RIPA lysis buffer (P0013B, Beyotime), and protein concentration was measured using the BCA Protein Assay Kit (BL521A, Biosharp). Equal amounts of protein were separated by SDS‐PAGE and transferred to PVDF membranes (ISEQ00010, Merck Millipore). After blocking with 5% skim milk, the membranes were incubated overnight at 4°C with primary antibodies against CFTR (Proteintech, 66928‐1‐Ig, 1:1000), YTHDF3 (Proteintech, 25537‐1‐AP, 1:1000), GAPDH (Proteintech, 60004‐1‐Ig, 1:5000), AMPKα1 (ABclonal, A1229, 1:1000) and phospho‐AMPKα1‐S496 (ABclonal, AP1002, 1:1000). The membranes were then incubated with HRP‐conjugated secondary antibodies, Goat anti‐Rabbit IgG‐HRP (BL003A, Biosharp, 1:5000) or Goat anti‐Mouse IgG‐HRP (BL001A, Biosharp, 1:5000), for 1 h at room temperature. Signals were developed using ECL chemiluminescent substrate (K‐12045‐D50, Advansta) and captured with a Chemiscope6100 imaging system (CLINX). Band intensity was measured using ImageJ. The intensity of each target protein was normalized to GAPDH, and the control group was set to 1. For AMPK activation analysis, p‐AMPK was normalized to total AMPK.

### 2.14 Cell Counting Kit 8 (CCK‐8) Assay

AC16 cell viability was evaluated with the CCK‐8 kit (CA1210, Solarbio). Log‐phase AC16 cells were digested with 0.25% trypsin (25200‐072, Gibco), resuspended in complete medium, and adjusted to 3 × 10^4^ cells/mL. Cells were seeded into a 96‐well plate at 100 μL (3 × 10^3^ cells) per well, with three technical replicate wells for each group. They were cultured in a 37°C, 5% CO_2_ incubator. After incubation, 10 μL of CCK‐8 solution was added to each well and incubated for 2 h, and absorbance was measured at 450 nm. The assay was performed in three independent biological experiments.

### 2.15 EdU

AC16 cell proliferation was measured using the BeyoClick EdU‐488 Cell Proliferation Detection Kit (C0071S, Beyotime). The procedure involved taking log‐phase AC16 cells, aspirating the culture medium and adding an equal volume of 2× EdU working solution (20 μM) to reach a final concentration of 10 μM, followed by a 2‐h incubation. Post‐incubation, the EdU solution was removed, and cells were fixed with 4% paraformaldehyde for 15 min at room temperature, then permeabilized with 0.3% Triton X‐100 in PBS for 10–15 min. Click reaction solution (50 μL) was added to each well, and cells were incubated in the dark at room temperature for 30 min, followed by three washes. DAPI solution was added, and cells were incubated in the dark for 10 min, followed by three more washes. Cells were then observed and imaged using a fluorescence microscope. EdU‐positive cells were counted from nine randomly selected fields in each group. The percentage of EdU‐positive cells was calculated as the number of EdU‐positive nuclei divided by the total number of DAPI‐stained nuclei.

### 2.16 Flow Cytometry

Apoptosis in AC16 cells was assessed using the Annexin V‐FITC Apoptosis Detection Kit (C1062S, Beyotime) and flow cytometry. Briefly, the culture medium was collected, and adherent cells were digested with EDTA‐free trypsin. Digestion was terminated with serum‐containing medium, and cells were collected as a single‐cell suspension. After centrifugation at 300 g for 5 min at 4°C, cells were washed twice with cold PBS and resuspended in 195 μL of 1× Binding Buffer at a concentration of 1–5 × 10^5^ cells/mL. Annexin V‐FITC (5 μL) and PI staining solution (10 μL) were added, and the cells were gently mixed and incubated for 10–20 min at room temperature in the dark. After staining, 400 μL of 1× Binding Buffer was added, and the samples were analysed by flow cytometry. At least 10,000 events were collected for each sample. Data were analysed using FlowJo software. Annexin V^−^/PI^−^ cells were considered viable cells, Annexin V^+^/PI^−^ cells were considered early apoptotic cells and Annexin V^+^/PI^+^ cells were considered late apoptotic cells. The total apoptotic rate was calculated as the sum of early and late apoptotic cells.

### 2.17 RNA Immunoprecipitation (RIP)‐qPCR

To detect the interaction between YTHDF3 and CFTR mRNA, we performed RIP‐qPCR experiments using the Methylated RNA Immunoprecipitation Kit (IEMed‐K305, IEMed Guangzhou Biomedical Technology Co. Ltd). Approximately 2 × 10^7^ cells were collected, and total RNA was extracted using Trizol lysis buffer. The RNA was then processed into fragments of about 100 nt through ultrasonication. A volume of 50 μL of RNA was taken as the Input group, while the remaining RNA was divided into IP and IgG groups, to which YTHDF3 antibody and IgG antibody were added, respectively, for immunoprecipitation. After the antibodies were bound to Protein A/G magnetic beads, the RNA was extracted following washing and elution steps. Finally, the enrichment of CFTR mRNA was detected by qPCR, using the following primers for amplification: CFTR‐F1: ATTTTGCATGAAGGTAGCAGC, CFTR‐R1: CCCCAAACTCTCCAGTCTGT; CFTR‐F2: AACTGCTGAACGAGAGGAGC, CFTR‐R2: CCATGAGCAAATGTCCCATGTC; CFTR‐F3: CATGGGACATTTGCTCATGGA, CFTR‐R3: AGTGTCCTCAATTCCCCTTACC.

### 2.18 RNA Stability Assay

For RNA stability analysis, AC16 cells were transfected with si‐YTHDF3 or negative control siRNA for 48 h, followed by treatment with 5 μM actinomycin D (HY‐17559, MCE) to block new RNA synthesis. Cells were collected at 0, 1, 2 and 4 h after actinomycin D treatment, and total RNA was extracted. CFTR mRNA levels were measured by RT‐qPCR as described above. The value at 0 h was set to 1, and the relative CFTR mRNA level at each time point was used to evaluate transcript stability. This assay was used to assess CFTR mRNA decay after transcriptional inhibition and was not intended to determine m6A modification of CFTR mRNA.

### 2.19 Construction of Rat Myocardial Ischemia–Reperfusion (I/R) Model

All animal protocols were approved by the Ethical Committee of Guangzhou Forevergen Medical Laboratory Animal Center (approval number: IACUC‐AEWC‐F250912001, dated September 12, 2025). Animal experiments were performed in accordance with the ARRIVE guidelines and the National Institutes of Health Guide for the Care and Use of Laboratory Animals. Twenty male Sprague–Dawley rats were randomly assigned to the sham, I/R, oeYTHDF3+I/R and oeYTHDF3+oeCFTR+I/R groups, with five rats in each group. Randomization was performed using a computer‐generated random number sequence. The sample size was determined based on preliminary experiments, commonly used group sizes in rat myocardial I/R studies, and ethical considerations to reduce animal use [18, 19]. A formal a priori power calculation was not performed, which should be considered a limitation of the in vivo design. Outcome analysis, including echocardiography, TTC quantification, histological assessment, immunofluorescence analysis and western blot quantification, was performed by investigators blinded to group allocation. For in vivo overexpression, rats in the oeYTHDF3 + I/R group received AAV‐YTHDF3 together with the corresponding control AAV, while rats in the oeYTHDF3+oeCFTR+I/R group received both AAV‐YTHDF3 and AAV‐CFTR. Rats in the sham and I/R groups received the same dose of empty AAV vector. The vectors were administered through tail vein injection at 1.0 × 10^12^ genome copies per rat, 4 weeks before I/R surgery. The myocardial I/R model was established by ligating the left anterior descending coronary artery for 30 min followed by 24 h of reperfusion. Briefly, rats were anaesthetised with isoflurane and intubated endotracheally. They were connected to a small animal ventilator for assisted respiration, and lead II electrocardiogram was recorded during surgery. A left thoracotomy was performed at the third‐fourth intercostal space to expose the heart. The left anterior descending coronary artery was ligated. Successful ischemia was confirmed by ST‐segment elevation and tall T waves on ECG, together with pale myocardial tissue below the ligation site. Reperfusion was confirmed by a reduction in ST‐segment elevation and gradual recovery of myocardial colour after the ligature was released. In the sham group, the suture was passed under the coronary artery without ligation. Samples were collected after 24 h of reperfusion for further analysis.

### 2.20 Echocardiographic Assessment of Cardiac Function in Rats

Echocardiography was used to assess cardiac function in rats. Before examination, rats were anaesthetised with isoflurane. Anaesthesia was induced with 5% isoflurane in oxygen for 2–3 min until the loss of the righting reflex and maintained with 1.5% isoflurane during image acquisition. Rats were placed in the supine position on a heating platform. Echocardiography was performed using a Mindray M90 SCI ultrasound system equipped with a rat cardiac probe. LVIDd, LVIDs, LVEF, LVFS and heart rate (HR) were measured from M‐mode images over three consecutive cardiac cycles. All measurements were obtained under the same anaesthetic conditions, and image analysis was performed by an investigator blinded to the experimental groups.

### 2.21 2,3,5‐Triphenyltetrazolium Chloride (TTC) Staining

After harvesting, heart tissues were immediately frozen at −20°C for 30 min, cut into 2–3 mm slices and incubated in 2% TTC staining solution (Solarbio, G3005) at 37°C for 15–30 min. After rinsing with PBS, the slices were fixed in 4% paraformaldehyde for 4–24 h and photographed. Infarct area was measured using ImageJ. Infarct size was expressed as infarct area/total ventricular slice area × 100%. Quantification was performed by an investigator blinded to group allocation.

### 2.22 Haematoxylin and Eosin (HE) Staining

Cardiac tissues were stained with HE staining solution (Servicebio, G1005) to analyse histological changes. Specific procedures were performed according to the manufacturer's instructions. Briefly, sections were placed in Haematoxylin staining solution for 3–5 min, followed by Haematoxylin differentiation solution for 2–5 s, and Haematoxylin bluing solution for 2–5 s. Water wash was performed after each step. Subsequently, sections were dehydrated sequentially in 85% and 95% graded ethanol for 5 min each, then stained in Eosin solution for 5 min. After dehydration twice in absolute ethanol for 5 min each, followed by dehydration in fresh absolute ethanol for 5 min, sections were cleared in xylene for 5 min and then cleared again in fresh xylene for another 5 min. After mounting with neutral balsam, morphological changes were observed under a microscope.

### 2.23 Immunofluorescence (IF) Detection of Caspase‐8 Expression in Cardiac Tissue

Cardiac tissue sections were baked at 60°C and then dewaxed and rehydrated through graded ethanol. After washing, antigen retrieval was performed by microwave heating in antigen retrieval buffer, and the sections were cooled to room temperature. The sections were blocked with 3% BSA for 1 h and incubated with caspase‐8 primary antibody (Proteintech, 13423‐1‐AP, 1:200) overnight at 4°C. After three washes with PBS, the sections were incubated with Alexa Fluor 488‐conjugated goat anti‐rabbit IgG H&L secondary antibody (Abcam, ab150077, 1:500) for 1 h at room temperature in the dark. Nuclei were stained with DAPI for 10 min. Finally, the sections were mounted with anti‐fade mounting medium (Biosharp, BL739B) and photographed using a Mshot PA53 FS6 SCAM fluorescence microscope. Fluorescence intensity was quantified using ImageJ from five randomly selected fields per sample.

### 2.24 ELISA Detection of Serum Cardiac‐Type Creatine Kinase (CK), Creatine Kinase Isoenzyme MB (CK‐MB), Lactate Dehydrogenase (LDH) and α‐Hydroxybutyrate Dehydrogenase (α‐HBDH)

Serum CK, CK‐MB, LDH and α‐HBDH levels were detected according to the kit instructions. CK kit (RX300944R), CK‐MB kit (RX301189R), LDH kit (RX300947R) and α‐HBDH kit (RX300219R) were purchased from Ruixin Biotech (Fujian, China).

### 2.25 Statistical Analysis

All statistical analyses were performed using GraphPad Prism 9.1. Data are presented as mean ± standard deviation (SD). In vitro experiments were performed with three independent biological replicates, and animal experiments included five rats per group. Technical replicates were performed where applicable and were not treated as independent biological samples. Data distribution and homogeneity of variance were checked before parametric tests. For comparisons between two groups, Student's *t*‐test was used. For comparisons among multiple groups, one‐way analysis of variance (ANOVA) was used, followed by Bonferroni test when variances were equal or Dunnett's T3 test when variances were unequal. *p* < 0.05 was considered statistically significant.

## 3 Results

### 3.1 CFTR as a Candidate Gene Identified From Myocardial Infarction‐Related Differential Expression Analysis

**FIGURE 1:**
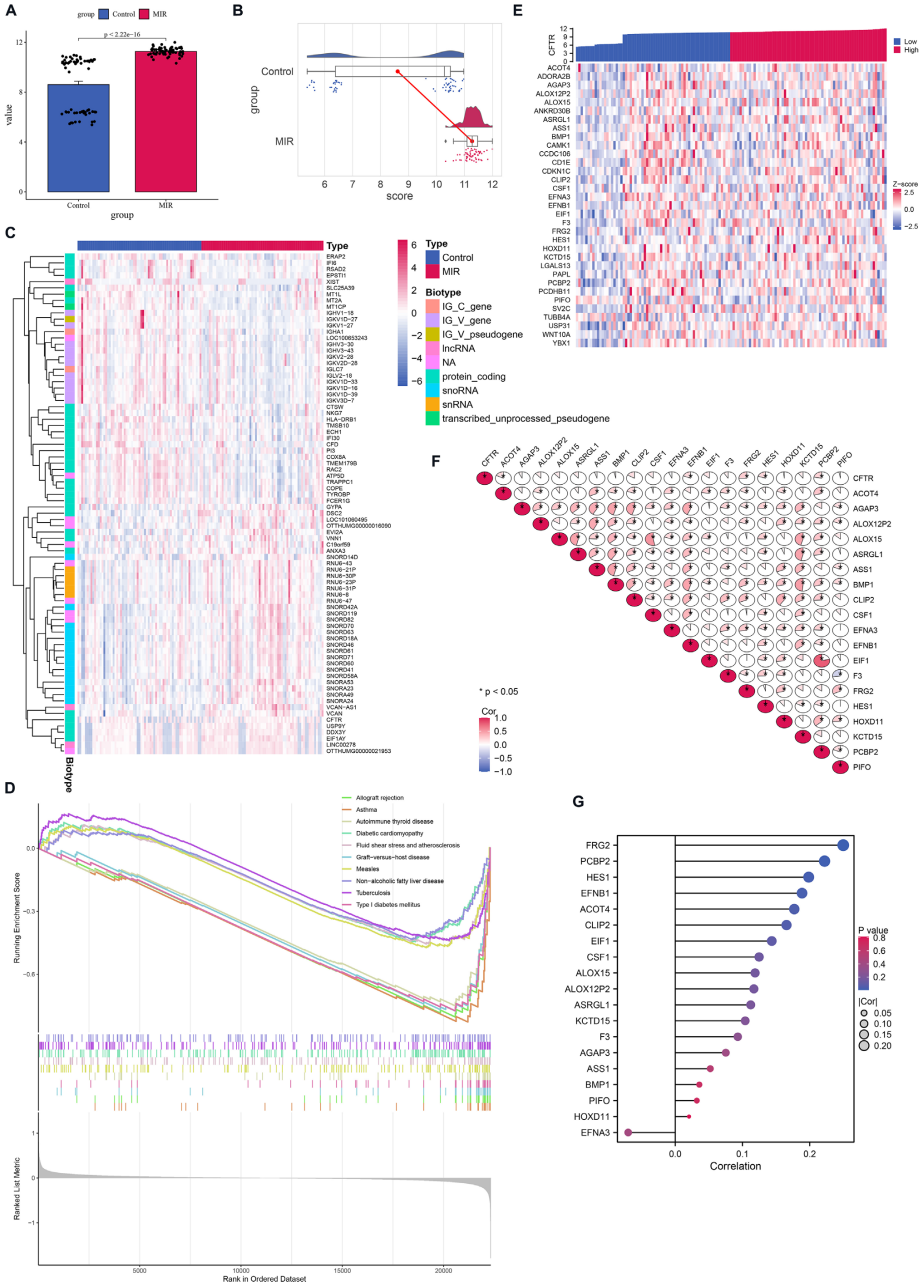
CFTR as a candidate gene identified from myocardial infarction‐related differential expression analysis. (A, B) Bar charts of CFTR expression in MIRI (A: Unmatched samples; B: Violin plot). (C) Clustered heatmap of DEGs related to MIRI and CFTR. (D) GSEA analysis of the GSE123342 dataset. (E–G) Heatmaps of CFTR‐related genes in MIRI.

Clinical and transcriptomic data associated with MIRI were obtained from the GEO website. Using R software, the clinical data were organized and analysed, revealing that CFTR was significantly upregulated in myocardial infarction‐related samples compared with stable coronary heart disease samples (Figure 1A,B). Using the criteria of adjusted *p* value < 0.05 and fold change ≥ 1.50‐fold (|log2FC| ≥ 0.58), 5444 DEGs were identified in the dataset and a heatmap of the MIRI‐CFTR‐related gene expression matrix was constructed (Figure 1C). After sorting the DEGs of MIRI by log2FC, GSEA revealed that in the GSE123342 dataset, the gene set for MIRI was significantly enriched in pathways such as Antigen Processing and Presentation, Allograft Rejection and Autoimmune Thyroid Disease (Figure 1D). Correlation analysis of the CFTR gene in MIRI revealed that, according to the screening criteria of *p* value_pearson < 0.05 and *p* value_spearman < 0.05, 6651 related genes were obtained. A co‐expression heatmap of CFTR was then constructed according to the high and low expression levels of CFTR gene (Figure 1E–G).

### 3.2 WGCNA of Core Genes and Enrichment Analysis

**FIGURE 2:**
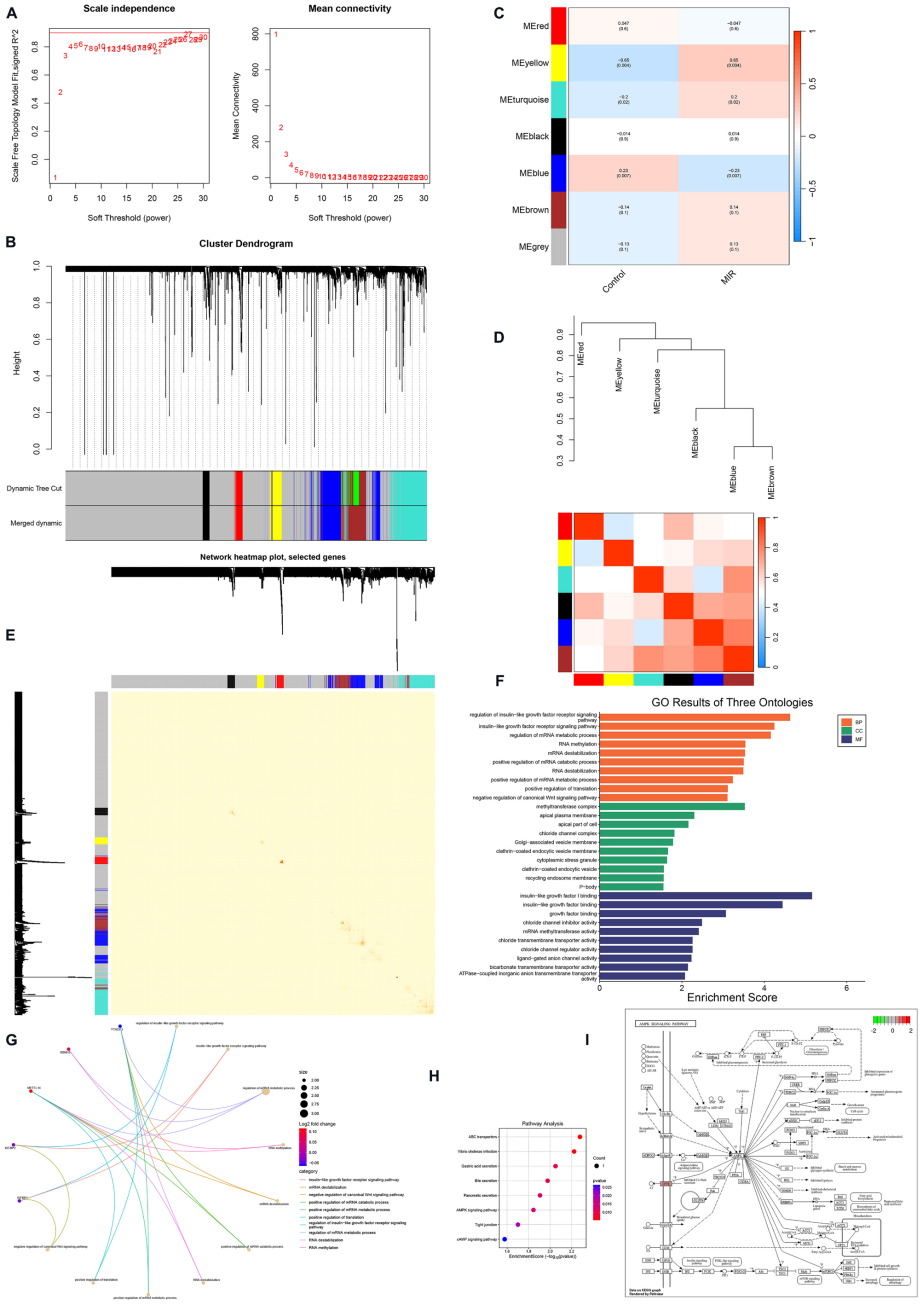
WGCNA of core genes and enrichment analysis. (A) Optimal soft threshold for WGCNA clustering. (B) Outlier analysis of samples. (C) Clustering tree of DEGs. (D) Relationships between the seven major modules and MIRI. (E) Heatmap of expression changes of co‐expressed genes in each cluster obtained from WGCNA clustering. (F, G) Bar chart chord diagram and of GO functional analysis. (H) Bubble chart of KEGG pathway enrichment analysis. (I) AMPK pathway.

The gene expression matrix of DEGs related to MIRI in the dataset was subjected to WGCNA. For the GSE123342 dataset, the value of soft threshold (*β*) was chosen as 27, at which scale‐free topology fitting index first exceeded 0.9 (Figure 2A). This result indicated that the dataset was suitable for further construction of a gene co‐expression network. The expression levels of 133 samples were used to calculate the correlation coefficients, followed by clustering analysis to measure any significant outlier samples. The results indicated that the 133 samples were well‐clustered with no obvious outliers, making them suitable for subsequent data analysis (Figure 2B). According to the soft threshold (*β*) = 27, a gene co‐expression network was constructed. TOM was calculated, and the gene dendrogram was constructed using the dissimilarity measure. Modules were identified by hybrid dynamic tree‐cutting method. All genes were divided into 7 modules (Figure 2C). Further correlation analysis between 7 gene co‐expression modules and MIRI microarray data revealed that the MEyellow module was significantly associated with MIRI (Figure 2D), with a correlation coefficient |*r*| ≥ 0.60 and *p* value < 0.05. This suggested that the MEyellow module may contain hub genes related to MIRI, and a total of 120 hub genes were identified. Finally, a heatmap of gene expression changes in each cluster obtained from the WGCNA clustering was constructed (Figure 2E). GO and KEGG pathway enrichment analyses were further performed on these hub genes. GO analysis of the potential target genes showed that their BP were primarily enriched in regulation of insulin‐like growth factor receptor signalling pathway, insulin‐like growth factor receptor signalling pathway, and regulation of mRNA metabolic process; their CC were primarily enriched in methyltransferase complex, apical plasma membrane and apical part of cell; and their MF were primarily enriched in insulin‐like growth factor I binding, insulin‐like growth factor binding and growth factor binding (Figure 2F,G). KEGG pathway enrichment analysis revealed that these genes were primarily concentrated in pathways including ABC transporters, AMPK pathway and cAMP signalling pathway (Figure 2H). Figure 2I further illustrated the AMPK pathway. Together, these enrichment results suggested that AMPK signalling may be related to the core genes identified in MIRI. This finding provided a basis for the following experimental analysis.

### 3.3 External Validation of Targets for MIRI

**FIGURE 3:**
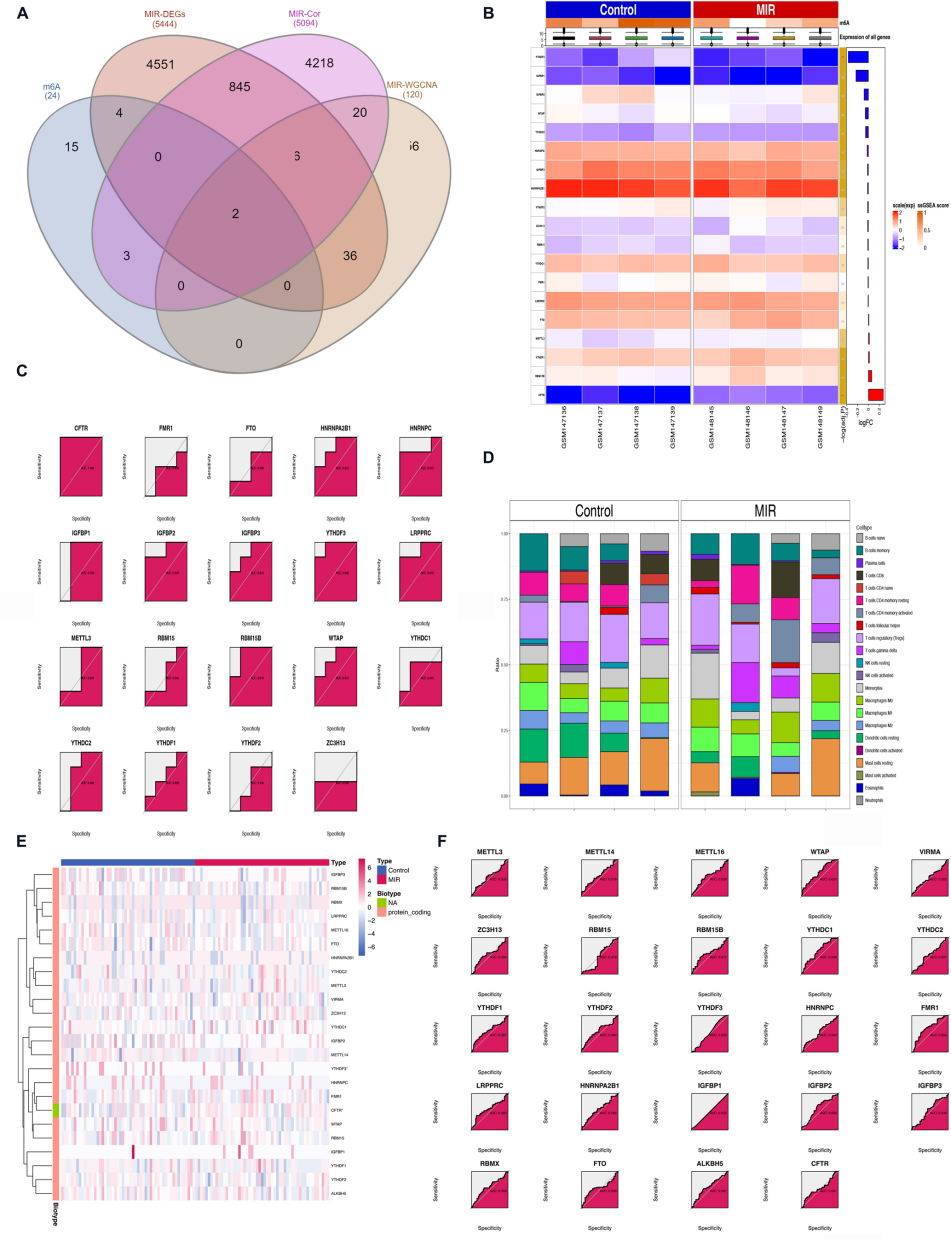
External validation of targets for MIRI. (A) Venn diagram of intersecting genes. (B) Heatmap of expression clustering of core genes in GSE6381 dataset. (C) ROC curves of DEGs. (D) Bar chart of immune cell distribution. (E) Heatmap of core gene expression in the GSE221740 dataset. (F) ROC curves of core DEGs in the GSE221740 dataset.

The 5444 DEGs related to CFTR treatment for MIRI, 6651 MIRI‐related genes, 120 MIRI‐WGCNA hub genes and 24 m6A genes obtained in a preliminary study were imported into the online Venn diagram tool InteractiVenn. After matching and mapping, two potential targets for CFTR treatment of MIRI were identified, including YTHDF3 and CFTR (Figure 3A). The screening criteria were set as follows: The keyword was ‘Myocardial Ischemia Reperfusion’; Human samples. The GSE6381 dataset, which included four disease‐related samples and four comparison samples, was used as a small external validation dataset. Using the same DEG screening standard, one upregulated DEG (CFTR) and one downregulated m6A‐related DEG (YTHDF3) were identified, and a heatmap of these genes was generated (Figure 3B). ROC curve analysis was performed to explore whether these genes could distinguish MIRI samples from the comparison samples in the analysed dataset. In the GSE6381 dataset, several genes, including CFTR and YTHDF3, showed AUC values above 0.80 (Figure 3C). However, this dataset included only four MIRI samples and four comparison samples. Therefore, these ROC results should be regarded as exploratory. Additionally, we applied the CIBERSORT tool to estimate the immune cell composition in MIRI and control tissues and analysed immune cell composition in each sample using this tool. Figure 3D showed the bar chart of immune cell distribution. Furthermore, all m6A genes were validated in the GSE221740 dataset, and we observed that CFTR expression was upregulated, while YTHDF3 expression was downregulated in myocardial ischemia–reperfusion injury, as shown in the heatmap of gene expression in Figure 3E. In the GSE221740 dataset, the AUC values of CFTR and YTHDF3 were only slightly above 0.50. Thus, these results were treated as descriptive observations rather than evidence of diagnostic relevance (Figure 3F).

### 3.4 Construction of the CFTR‐Transcription Factor Regulatory Network and Validation of Expression Levels

**FIGURE 4:**
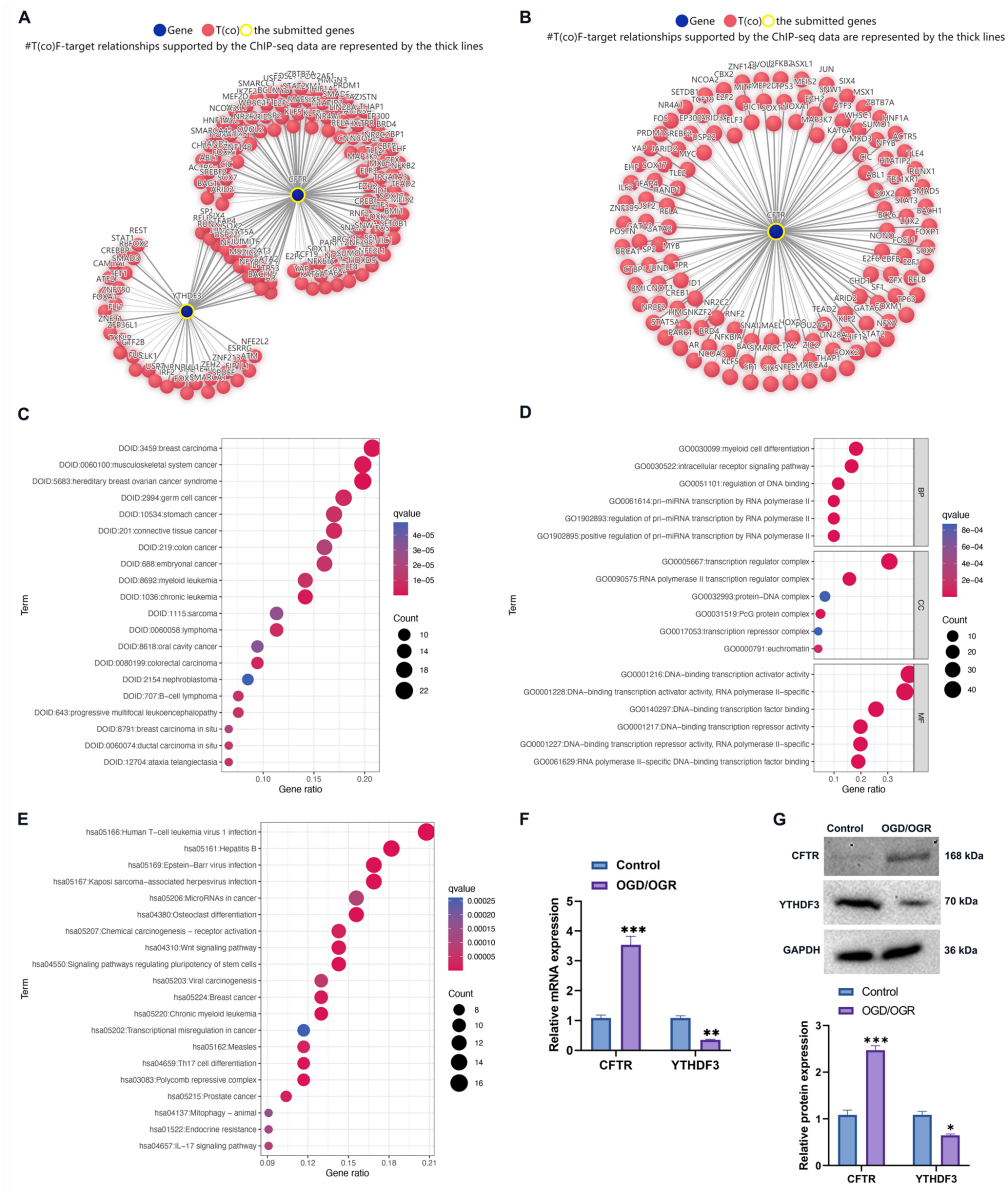
Construction of the CFTR‐transcription factor regulatory network and validation of expression levels. (A) Core transcription factor regulatory network. (B) CFTR‐transcription factor regulatory network. (C–E) DO, GO and KEGG enrichment analysis of targets related to CFTR transcription factors. (F) RT‐qPCR detection of CFTR and m6A regulator YTHDF3 expression (*N* = 3 independent biological replicates). (G) Western blot detection of CFTR and m6A regulator YTHDF3 expression (*N* = 3 independent biological replicates). *p < 0.05, **p < 0.01, ***p < 0.001.

The two core genes (YTHDF3 and CFTR) mentioned above were input into the KnockTF database to retrieve the transcription factors associated with these core genes, identifying 155 transcription factors with the highest relevance. Figure 4A displayed the core gene‐transcription factor regulatory PPI network. Further analysis of the core gene CFTR revealed its close association with 124 transcription factors, including TP53, STAT3 and TFAP4, with the PPI network shown in Figure 4B. DO/GO/KEGG enrichment analysis was performed on the potential target sites of CFTR's transcription factors. The DO analysis identified that four genes were enriched in acute myocardial infarction, including HIF1A, POSTN, SOX2 and TP53 (Figure 4C). GO analysis indicated that these genes were mainly enriched in positive regulation of pri‐miRNA transcription by RNA polymerase II, intracellular receptor signalling pathway and regulation of pri‐miRNA transcription by RNA polymerase II (Figure 4D). KEGG analysis revealed enrichment in pathways including Human T‐cell leukaemia virus 1 infection, Hepatitis B, Chronic myeloid leukaemia and Wnt signalling pathway (Figure 4E). Subsequently, we constructed an OGD/OGR model using AC16 cells. We found that compared to the Control group, CFTR mRNA and protein levels were remarkably elevated, while YTHDF3 mRNA and protein levels were notably reduced in the OGD/OGR group (Figure 4F,G).

### 3.5 Overexpression of YTHDF3 Aggravated Cell Injury in the OGD/OGR Model

**FIGURE 5:**
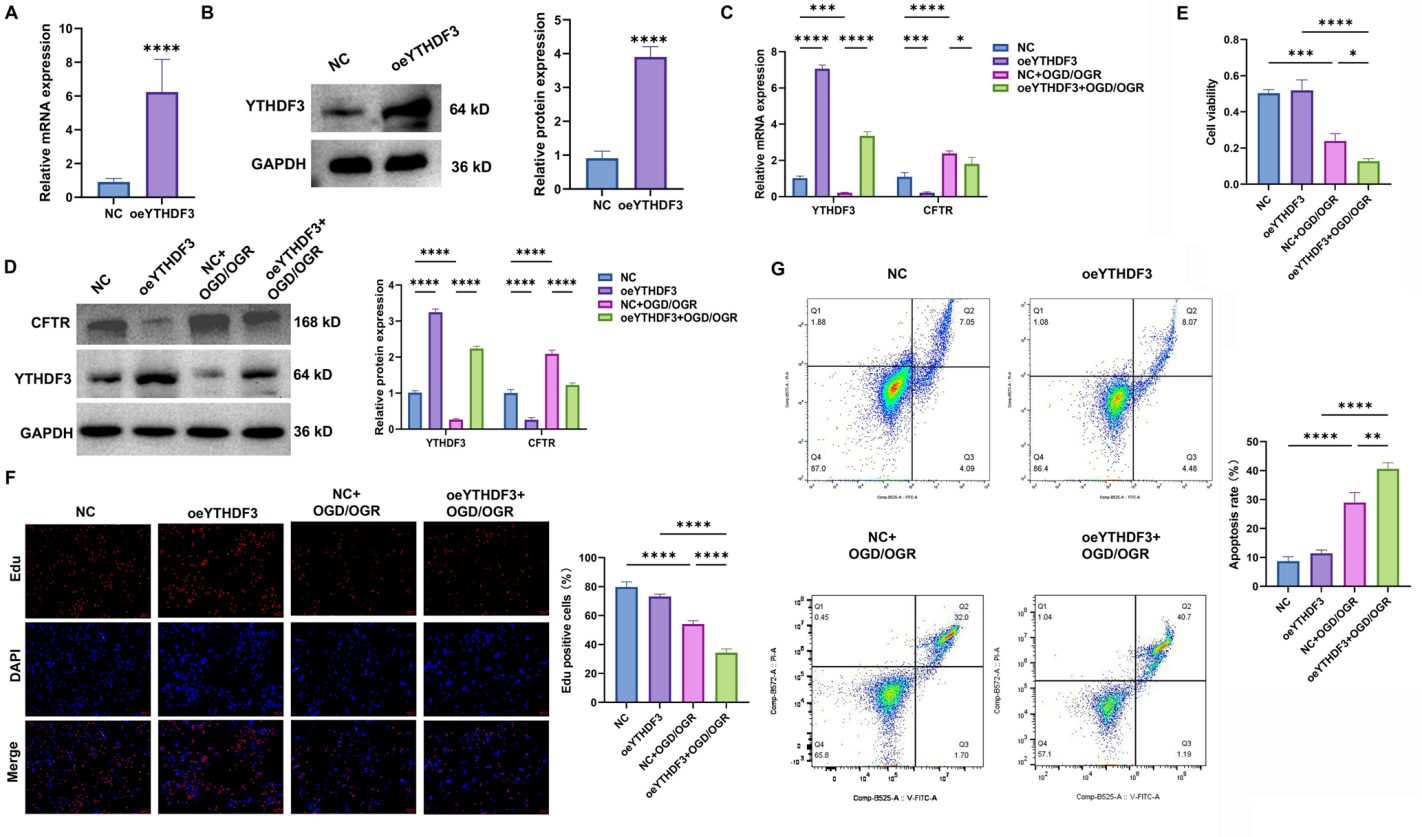
Overexpression of YTHDF3 aggravated cell injury in the OGD/OGR model. (A, B) YTHDF3 levels measured by RT‐qPCR and Western blot after overexpression of YTHDF3. (C, D) YTHDF3 and CFTR levels assessed by RT‐qPCR and western blot after overexpression of YTHDF3 and establishment of the OGD/OGR model. (E–G) Cell viability, proliferation, and apoptosis determined by CCK‐8 assay, EdU staining and flow cytometry after overexpression of YTHDF3 and establishment of the OGD/OGR model. *N* = 3 independent biological replicates. *p < 0.05, **p < 0.01, ***p < 0.001, ****p < 0.0001.

Next, we constructed YTHDF3 overexpression plasmids. In comparison with the NC group, YTHDF3 mRNA and protein levels were substantially increased in the oeYTHDF3 group (Figure 5A,B). After overexpressing YTHDF3 and establishing the OGD/OGR model, we found that relative to the NC group, YTHDF3 levels were remarkably raised, while CFTR levels were markedly reduced in the oeYTHDF3 group. However, YTHDF3 levels were notably repressed, and CFTR levels were markedly elevated in the NC+OGD/OGR group. In contrast with the NC+OGD/OGR group, YTHDF3 was considerably elevated and CFTR was notably repressed in the oeYTHDF3+OGD/OGR group (Figure 5C,D). Cell function experiments indicated that in comparison with the NC group, the viability and proliferation abilities of AC16 cells were remarkably suppressed, and the apoptosis was substantially increased in the NC+OGD/OGR group. In contrast with the NC+OGD/OGR group, the viability and proliferation abilities of AC16 cells were further suppressed, and the apoptosis was further increased in the oeYTHDF3+OGD/OGR group (Figure 5E–G). To further investigate the role of YTHDF3, we next examined the effects of YTHDF3 knockdown in the OGD/OGR model. As shown in Figure S1, YTHDF3 knockdown in AC16 cells resulted in a significant decrease in YTHDF3 expression, both at the mRNA and protein levels (Figure S1A,B). In contrast to the oeYTHDF3 + OGD/OGR group, the cell viability, proliferation and apoptosis of siYTHDF3 + OGD/OGR cells were also significantly altered (Figure S1C–E). Together, these results suggest that YTHDF3 overexpression reduces CFTR expression and aggravates OGD/OGR‐induced injury in AC16 cells.

### 3.6 CFTR Overexpression Partly Attenuated YTHDF3‐Associated Injury in the OGD/OGR Model

**FIGURE 6:**
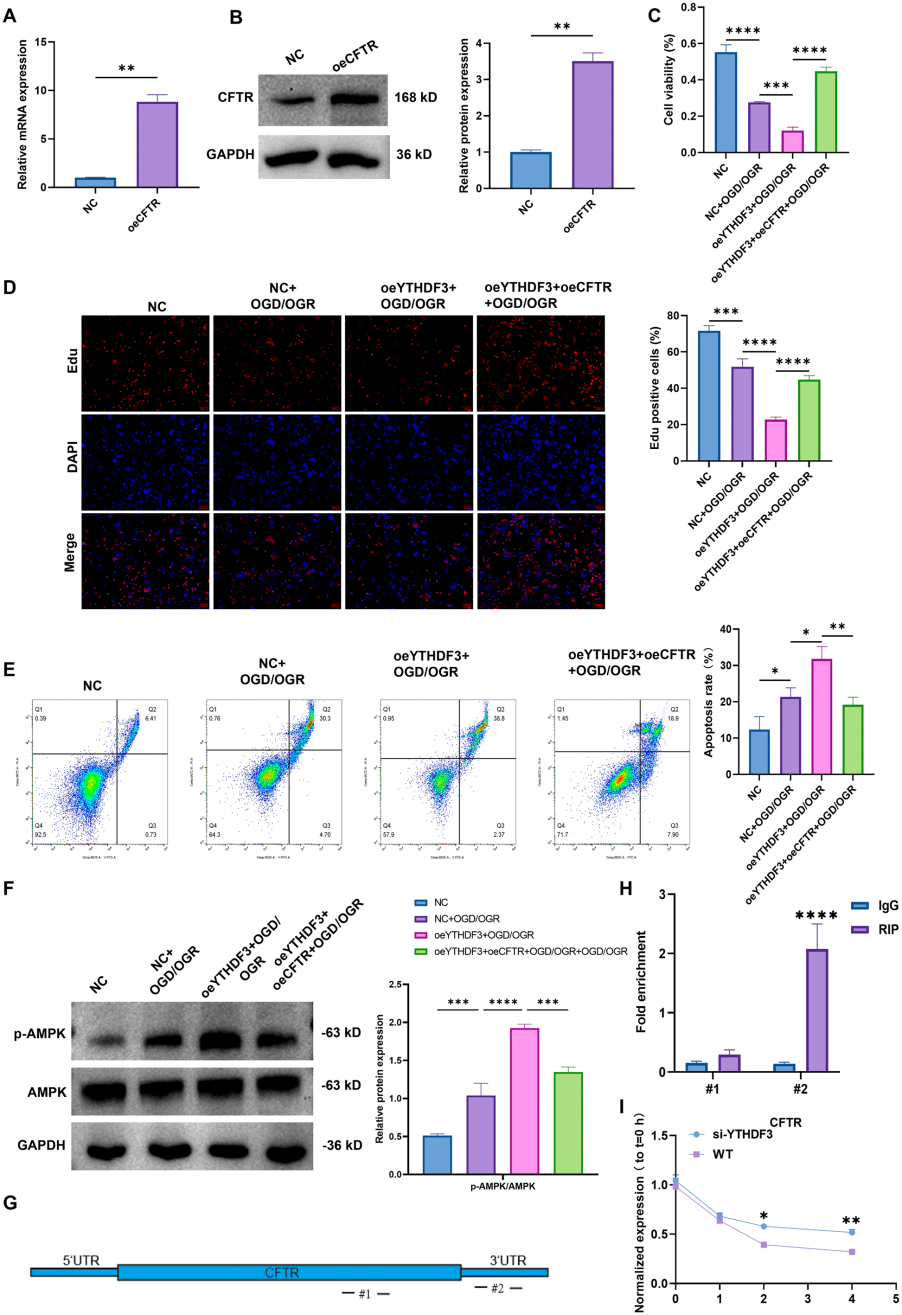
CFTR overexpression partly attenuated YTHDF3‐associated injury in the OGD/OGR model. (A) RT‐qPCR analysis of CFTR mRNA expression following overexpression of CFTR. (B) Western blot analysis showing CFTR protein expression after overexpression of CFTR. (C–E) Cell viability, proliferation and apoptosis assessed by CCK‐8 assay, EdU staining and flow cytometry after overexpression of YTHDF3 and CFTR and establishment of the OGD/OGR model. (F) Western blot analysis of AMPK and p‐AMPK expression. (G) Schematic diagram of the potential binding sites of YTHDF3 and CFTR. H. Interaction between YTHDF3 and CFTR assessed by RIP‐qPCR. I. CFTR mRNA stability was assessed by actinomycin D chase assay. AC16 cells were treated with 5 μM actinomycin D, and RNA was collected at 0, 1, 2 and 4 h for RT‐qPCR analysis. CFTR mRNA levels were normalized to the level at 0 h. *N* = 3 independent biological replicates. *p < 0.05, **p < 0.01, ****p < 0.0001.

Based on the results in Figure 5, YTHDF3 overexpression reduced CFTR expression under OGD/OGR conditions. We then overexpressed CFTR to examine whether increased CFTR expression could attenuate the effects associated with YTHDF3 overexpression. CFTR overexpression was first confirmed at both the mRNA and protein levels (Figure 6A,B). Cell function experiments further indicated that, in contrast with the NC group, the viability and proliferation abilities of AC16 cells were markedly suppressed and apoptosis was notably increased in the NC + OGD/OGR group. After further overexpression of YTHDF3, cell viability and proliferation were further decreased, while apoptosis was further increased. However, additional CFTR overexpression partly attenuated the changes in cell viability, proliferation and apoptosis observed after YTHDF3 overexpression (Figure 6C–E). Western blot analysis showed that YTHDF3 overexpression further increased the p‐AMPK/AMPK ratio under OGD/OGR conditions, whereas CFTR overexpression reduced this increase in the oeYTHDF3+oeCFTR+OGD/OGR group (Figure 6F). Figure 6G shows the predicted binding regions between YTHDF3 and CFTR mRNA. RIP‐qPCR showed that CFTR mRNA was enriched in the YTHDF3 immunoprecipitated fraction compared with the IgG control, suggesting an association between YTHDF3 and CFTR mRNA (Figure 6H). To further examine whether YTHDF3 affected CFTR transcript turnover, an actinomycin D chase assay was performed. YTHDF3 knockdown slowed the decay of CFTR mRNA compared with the negative control group, suggesting that YTHDF3 may affect CFTR transcript stability (Figure 6I). These results support a possible post‐transcriptional relationship between YTHDF3 and CFTR, but they do not establish m6A‐dependent regulation. To investigate the effects of YTHDF3 and CFTR knockdown on cell function, AC16 cells were transfected with si‐YTHDF3 and si‐CFTR and then subjected to OGD/OGR treatment. YTHDF3 knockdown increased cell viability (Figure S2A) and proliferation (Figure S2B), while reducing apoptosis (Figure S2C). However, these effects were partially reversed by CFTR knockdown. Together, these findings suggest that CFTR expression may be involved in YTHDF3‐associated cellular responses under OGD/OGR conditions.

### 3.7 CFTR Knockdown Affected Cell Viability, Proliferation, Apoptosis, and AMPK Signalling in AC16 Cells

**FIGURE 7:**
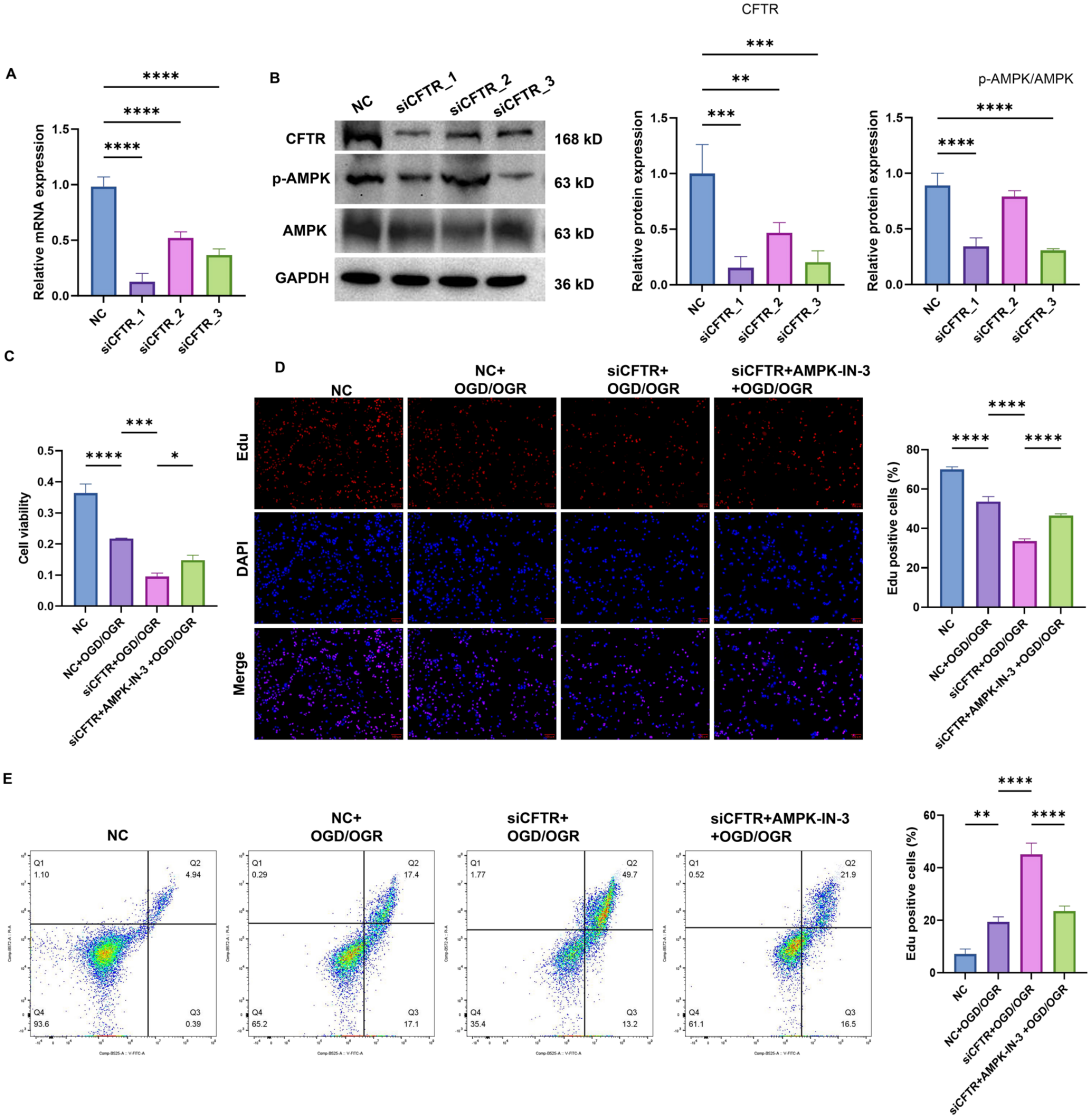
Effects of CFTR knockdown and AMPK inhibition on cell function and CFTR expression in AC16 cells. (A) CFTR mRNA expression was measured by RT‐qPCR after transfection with si‐CFTR_1, si‐CFTR_2 and si‐CFTR_3. (B) CFTR and p‐AMPK/AMPK protein levels were confirmed by Western blot analysis after siRNA‐mediated CFTR knockdown. (C–E) Cell viability, proliferation, and apoptosis were evaluated using CCK‐8, Edu staining and flow cytometry after transfection with si‐CFTR and subsequent OGD/OGR treatment, with or without AMPK‐IN‐3 inhibition. *N* = 3 independent biological replicates. *p < 0.05, **p < 0.01, ***p < 0.001, ****p < 0.0001.

To investigate the role of CFTR knockdown and AMPK inhibition in AC16 cells, the cells were transfected with si‐CFTR and subjected to OGD/OGR treatment with or without AMPK‐IN‐3 intervention. RT‐qPCR data denoted that CFTR mRNA expression was decreased in all si‐CFTR groups (siCFTR_1, siCFTR_2 and siCFTR_3) compared to the NC group, with siCFTR_1 and 3 showing the most significant reduction (Figure 7A). Western blot analysis revealed that CFTR protein expression was lower in the siCFTR groups, and the p‐AMPK/AMPK ratio was also reduced in these groups compared to the NC group (Figure 7B). Cell viability, measured by CCK‐8, was decreased in the NC+OGD/OGR group compared to the NC group; the siCFTR+OGD/OGR group showed further reduction in cell viability, while the siCFTR+OGD/OGR+AMPK‐IN‐3 group exhibited significant improvement in cell viability (Figure 7C). Similarly, Edu staining revealed that CFTR knockdown reduced the percentage of Edu‐positive cells in the AC16 cells of the OGD/OGR model, while AMPK‐IN‐3 intervention increased this percentage, indicating enhanced proliferation (Figure 7D). Meanwhile, CFTR knockdown increased apoptosis in AC16 cells under the OGD/OGR model, while AMPK‐IN‐3 intervention partially reversed this effect (Figure 7E). These results suggest that CFTR knockdown aggravates OGD/OGR‐induced cellular injury, while AMPK‐IN‐3 partially improves cell viability, proliferation and apoptosis under these conditions. However, the relationship between CFTR expression, p‐AMPK/AMPK changes and the effect of AMPK‐IN‐3 requires further investigation.

### 3.8 CFTR Overexpression Attenuated YTHDF3‐Associated I/R Injury in Rats

**FIGURE 8:**
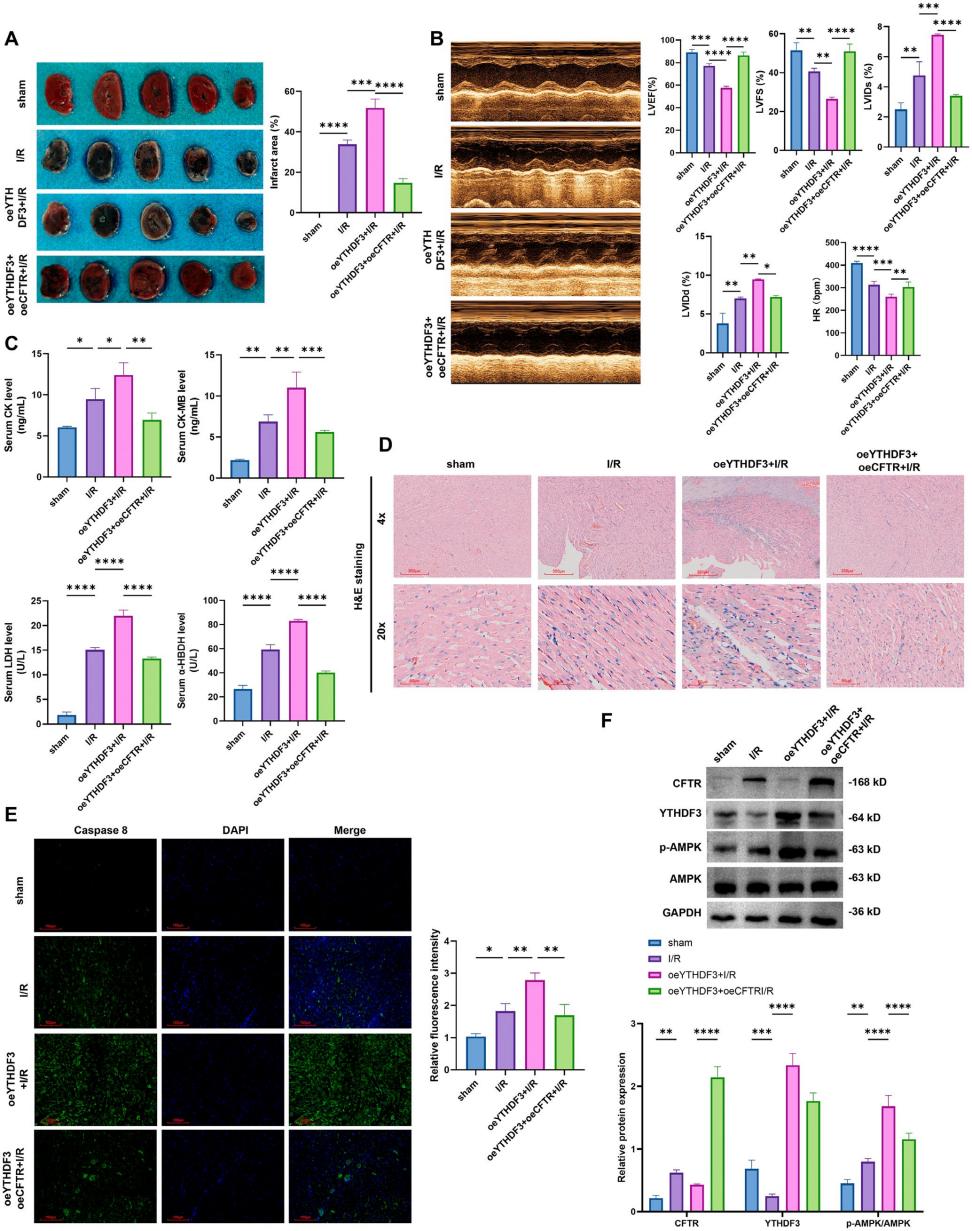
CFTR overexpression attenuated YTHDF3‐associated I/R injury in rats. (A) Myocardial infarct area determined by TTC staining. (B) Cardiac function assessed by echocardiography, including LVEF, LVFS, LVIDs, LVIDd and HR. (C) Serum CK, CK‐MB, LDH and α‐HBDH levels detected by ELISA kit. (D) Cardiac injury evaluated by HE staining. (E) The apoptotic marker caspase‐8 in cardiac tissue assessed by IF detection. (F) CFTR, YTHDF3, p‐AMPK and AMPK expressions were determined by western blot analysis. *n* = 5 rats per group; Western blot analysis was performed using *n* = 3 biological samples per group. *p < 0.05, **p < 0.01, ***p < 0.001.

TTC staining results displayed that the myocardial infarct size was significantly increased in the I/R group compared with the sham group, and overexpression of YTHDF3 further increased the infarct size. However, CFTR overexpression reduced the increase in cardiac injury observed in the oeYTHDF3+I/R group (Figure 8A). Echocardiography results showed that compared with the sham group, LVEF, LVFS, and HR were decreased while LVIDs and LVIDd were increased in the I/R group. Overexpression of YTHDF3 further decreased LVEF, LVFS and HR and increased LVIDs and LVIDd. However, compared with the oeYTHDF3+I/R group, LVEF, LVFS and HR were significantly upregulated while LVIDs and LVIDd were significantly downregulated in the oeYTHDF3+oeCFTR+I/R group (Figure 8B). Serum ELISA results showed that compared with the sham group, serum levels of CK, CK‐MB, LDH and α‐HBDH were significantly upregulated in the I/R group, and overexpression of YTHDF3 further increased their secretion; however, compared with the oeYTHDF3+I/R group, the expression levels of CK, CK‐MB, LDH and α‐HBDH were significantly downregulated in the oeYTHDF3+oeCFTR+I/R group (Figure 8C). Similarly, HE staining showed that compared with the sham group, cardiomyocytes in the I/R group exhibited pyknosis, fragmentation and even dissolution and disappearance of nuclei. The cytoplasm appeared dark red due to enhanced eosinophilia, with blurred or lost striations. Cells were disarranged, and a large number of inflammatory cells were visible in the interstitium. Compared with the I/R group, myocardial injury was aggravated with more inflammatory cells in the oeYTHDF3+I/R group; compared with the oeYTHDF3+I/R group, myocardial injury was alleviated in the oeYTHDF3+oeCFTR+I/R group (Figure 8D). IF showed that compared with the sham group, the expression level of caspase‐8 was significantly upregulated in the I/R group, and overexpression of YTHDF3 further increased caspase‐8 expression; compared with the oeYTHDF3+I/R group, the expression level of caspase‐8 was significantly downregulated in the oeYTHDF3+oeCFTR+I/R group (Figure 8E). Furthermore, western blot analysis showed that, compared with the sham group, CFTR expression and the p‐AMPK/AMPK ratio were increased in the I/R group, while YTHDF3 expression was decreased. Compared with the I/R group, YTHDF3 overexpression increased YTHDF3 expression and further increased the p‐AMPK/AMPK ratio, while CFTR expression was decreased in the oeYTHDF3+I/R group. Compared with the oeYTHDF3+I/R group, additional CFTR overexpression increased CFTR expression and reduced the p‐AMPK/AMPK ratio in the oeYTHDF3+oeCFTR+I/R group (Figure 8F). These results suggest that YTHDF3‐related changes in I/R injury may be associated with CFTR expression.

## 4 Discussion

This study suggests that YTHDF3 may be associated with MIRI and CFTR‐related changes. By combining bioinformatics analysis with cellular and animal experiments, our findings point to a possible relationship between YTHDF3 and CFTR, along with changes in AMPK signalling. However, these data do not establish a direct mechanistic pathway, and further validation is still needed.

Bioinformatics analysis is useful for screening disease‐related genes and identifying possible regulatory clues from public datasets [20, 21, 22]. In MIRI research, it has been used to mine DEGs and predict pathways that may be worth further testing [23]. In this study, we analysed the GSE123342 dataset and identified 5444 DEGs, among which CFTR was significantly upregulated. Because this workflow combined DEG analysis, WGCNA, correlation analysis, disease‐related databases, and m6A‐related gene lists, the bioinformatics results should be viewed as hypothesis‐generating rather than direct mechanistic evidence. CFTR is an important membrane protein primarily involved in chloride ion transport across the membrane, maintaining ion balance inside and outside the cell [24]. It is instrumental in a range of physiological and pathological processes, such as regulating intracellular chloride ion concentration, maintaining cell volume and function [25]. In MIRI, CFTR expression may be related to changes in myocardial cell survival and functional status. However, the specific role of CFTR in MIRI and its regulatory mechanisms have not been fully elucidated. The identification of CFTR upregulation in myocardial infarction‐related samples and experimental I/R models provides a basis for further investigation of its potential role in myocardial injury.

Through WGCNA, we identified 120 hub genes in the MEyellow module, which was associated with MIRI under the preset filtering criteria. KEGG enrichment analysis showed enrichment in pathways such as ABC transporters and the AMPK pathway. These results provide possible biological clues, but they should be interpreted as pathway‐level associations rather than proof of a direct mechanism. ABC transporters are involved in the transmembrane transport of various substances, and their dysfunction can lead to intracellular homeostasis disruption, exacerbating myocardial injury [26]. AMPK pathway is pivotal in regulating cellular energy metabolism, oxidative stress responses and cell survival, with its dysregulation being strongly associated with the development of various cardiovascular diseases [27, 28]. Prior research has indicated that activating the AMPK pathway could alleviate MIRI [29]. The enrichment of related genes in this pathway suggests that AMPK signalling may be related to CFTR‐associated changes in MIRI.

In the GSE6381 dataset, CFTR and YTHDF3 expression levels differed between groups, and ROC analysis showed some separation in this small dataset. However, because the dataset included only four MIRI samples and four comparison samples, this result should be viewed as a preliminary observation. It is not sufficient to support a diagnostic claim. Currently, MIRI diagnosis primarily relies on clinical symptoms, electrocardiograms and myocardial enzyme markers [30], which have certain limitations. These expression changes may provide a basis for future studies with larger sample sizes. The association of CFTR with transcription factors such as TP53, STAT3 and TFAP4 suggests that CFTR may be involved in a broader transcriptional regulatory network in cardiomyocytes. TP53, a tumour suppressor gene, is crucial for apoptosis and cell cycle regulation [31]. Previous evidence has shown that TP53 is involved in the pathological process of MIRI [32]. STAT3, which regulates inflammatory responses and cell survival, also significantly impacts MIRI [33]. The interaction between these transcription factors and CFTR may be involved in MIRI progression and may provide clues for further mechanistic studies. The Wnt signalling pathway was also enriched among the predicted targets of CFTR‐related transcription factors. This result may be relevant to cardiac repair after ischemic injury. Previous work showed that inhibition of the canonical Wnt pathway reduced post‐ischemic remodelling and helped preserve cardiac function [34]. Thus, the Wnt enrichment observed here may suggest a link between the CFTR‐related transcriptional network and myocardial remodelling after I/R injury. Since Wnt signalling was not directly tested in this study, this finding remains exploratory. m6A reader proteins (such as the YTHDF family), as core elements of post‐transcriptional regulation, may mediate target gene expression by recognizing m6A modification sites [35, 36], but their role in MIRI has not yet been systematically explored. In the present study, RIP‐qPCR showed enrichment of CFTR mRNA in the YTHDF3 immunoprecipitated fraction, and the actinomycin D chase assay suggested that YTHDF3 knockdown slowed CFTR mRNA decay. These findings support a possible post‐transcriptional relationship between YTHDF3 and CFTR. However, they do not directly demonstrate that CFTR mRNA is m6A‐modified or that the binding of YTHDF3 to CFTR mRNA is m6A‐dependent. Because MeRIP‐qPCR, mutation of predicted m6A sites and translation‐efficiency assays were not performed, this result should be interpreted as preliminary evidence for YTHDF3‐associated regulation of CFTR mRNA rather than proof of an m6A‐dependent mechanism.

In both the OGD/OGR model and the rat myocardial I/R model, YTHDF3 expression was significantly reduced, while CFTR expression was increased. In the rat myocardial I/R model, YTHDF3 overexpression worsened myocardial injury. This was consistent with the in vitro findings, where YTHDF3 overexpression was accompanied by lower CFTR expression, increased apoptosis and reduced proliferation. In contrast, CFTR overexpression partly attenuated these changes in the I/R model. These findings suggest that CFTR may be involved in the regulation of cell survival and apoptosis during ischemia–reperfusion injury. In the cell model, YTHDF3 knockdown also affected injury‐related cell phenotypes. Together with the rat overexpression data, these results support a possible role of YTHDF3 in myocardial I/R injury. The OGD/OGR experiments further suggest that changes in CFTR expression may affect cellular responses related to YTHDF3 regulation. These findings suggest that the YTHDF3‐CFTR relationship may be involved in the cellular response to ischemia–reperfusion stress. In our acute model, CFTR overexpression did not worsen cellular injury and instead showed a protective trend. This may reflect a compensatory change under I/R‐related stress, but this explanation still needs further testing. CFTR is involved in chloride and fluid transport, which may be related to ion balance and edema changes during ischemia–reperfusion injury [37, 38]. However, we recognize that prolonged CFTR overexpression could have different outcomes in chronic conditions, potentially leading to maladaptive effects. These findings suggest that CFTR‐related changes may be linked to the injury response in the acute model.

From a biological perspective, CFTR, as a member of the ABC transporter family and a cAMP‐activated chloride channel, is associated with various cardiovascular diseases when its function is impaired [38, 39]. Its upregulation in MIRI may be related to changes in ionic homeostasis, cell volume regulation, or inflammatory responses. As an m6A reader, YTHDF3 typically functions by promoting mRNA degradation or inhibiting translation [40]. The negative relationship between YTHDF3 and CFTR suggests that reduced YTHDF3 under I/R stress may be accompanied by increased CFTR expression. This increase may reflect a compensatory change, but its role and duration‐dependent effects remain unclear. These results suggest a possible link among RNA regulation, ion‐channel‐related changes and metabolic signalling in MIRI. KEGG enrichment analysis showed enrichment of the AMPK pathway, suggesting that AMPK signalling may be related to the YTHDF3‐CFTR‐associated response to ischemia–reperfusion stress. In our experiments, YTHDF3 overexpression and CFTR modulation were accompanied by changes in the p‐AMPK/AMPK ratio. AMPK‐IN‐3 also partially improved the cellular injury caused by CFTR knockdown under OGD/OGR conditions. These findings suggest that AMPK‐related signalling may participate in the injury phenotype observed in this model. However, AMPK activation should not be interpreted as uniformly protective or detrimental, because its role may vary with the stage of ischemia–reperfusion and the cellular context [41]. A possible link between CFTR and AMPK may involve ionic or metabolic stress. CFTR is involved in chloride transport, and chloride‐channel‐related changes have been linked to mitochondrial structure and function [42]. AMPK is sensitive to cellular energy status and responds to changes in AMP/ATP and ADP/ATP ratios [43]. AMPK‐related metabolic changes have also been reported in CFTR‐dysfunctional models [44]. Nevertheless, chloride homeostasis, mitochondrial function, ATP levels, AMP/ATP ratio and autophagic flux were not directly examined in this study. Therefore, the current data should be interpreted as AMPK‐associated signalling changes rather than evidence that CFTR directly regulates AMPK.

Taken together, the RIP‐qPCR and actinomycin D chase assays support a possible post‐transcriptional link between YTHDF3 and CFTR. However, this evidence should not be interpreted as proof of m6A‐dependent regulation. Direct confirmation will require MeRIP‐qPCR, functional testing of predicted m6A sites and assays evaluating CFTR translation efficiency. This study provides preliminary evidence linking YTHDF3 with CFTR‐related changes in MIRI. Current research indicates that YTHDF family members influence disease progression in tumours and neurodegenerative diseases by modulating the translation of target genes [45], but their functions in MIRI have not been clarified. One possible explanation is that the decrease in YTHDF3 under I/R stress may represent a compensatory response, which could allow higher CFTR expression. This remains a hypothesis and needs to be tested in future studies. This may provide a useful clue for further studying m6A‐related regulation in MIRI. Our findings suggest that CFTR expression may be influenced by post‐transcriptional regulation in MIRI, but whether m6A modification is directly involved remains unresolved. The expression differences observed in these datasets need to be tested in larger and independent cohorts before any biomarker relevance can be considered. Future studies should first clarify how AMPK signalling is related to the YTHDF3‐CFTR‐associated response in MIRI.

Although this study suggests a possible relationship between YTHDF3, CFTR and MIRI, several limitations remain. The bioinformatics analysis included several public datasets, databases and filtering steps. Although the thresholds were prespecified, these findings should be viewed as hypothesis‐generating and need further validation in independent datasets and experimental models. A major limitation is that this study did not directly verify m6A modification of CFTR mRNA. Although RIP‐qPCR and the actinomycin D chase assay suggested that YTHDF3 may associate with CFTR mRNA and affect its stability, these assays cannot determine whether this regulation depends on m6A. Future studies using MeRIP‐qPCR, m6A‐site mutagenesis and translation‐related assays are needed to clarify whether CFTR is directly regulated through an m6A‐dependent mechanism. Another limitation is that AMPK signalling was assessed mainly by p‐AMPK/AMPK changes and AMPK‐IN‐3 intervention. We did not measure chloride homeostasis, mitochondrial function, ATP levels, AMP/ATP ratio, or autophagic flux. Therefore, the relationship between CFTR expression and AMPK signalling remains provisional and requires further mechanistic testing. Additionally, while this study focused on YTHDF3 and CFTR in MIRI, further research is needed to investigate the roles of other m6A regulators and their target genes in this context. The rat MIRI model supported the in vitro findings, but MIRI is a complex systemic condition. Further studies using additional models will be needed to better understand the possible YTHDF3‐CFTR relationship in the broader context of myocardial injury. Only male rats were used in this study. This design helped reduce sex hormone‐related variation in the initial in vivo experiments, but the findings may not fully apply to female animals. Female sex hormones and estrous cycle status may affect myocardial I/R injury, as infarct size has been reported to differ across estrous stages in female rats [46]. Studies including female rats will be needed to further examine the possible YTHDF3‐CFTR relationship. The ROC analysis also has clear limitations. The GSE6381 dataset included only four MIRI samples and four comparison samples, and the AUC values in another dataset were only slightly above 0.50. Therefore, these findings should be regarded as preliminary expression‐based observations rather than evidence of diagnostic utility. Larger independent cohorts are needed before their biomarker relevance can be assessed.

## 5 Conclusion

In summary, this study suggests a possible relationship between YTHDF3 and CFTR in MIRI. In the cell and rat I/R models, YTHDF3 overexpression was associated with lower CFTR expression and more severe injury, while CFTR overexpression partly attenuated these changes. RIP‐qPCR and the actinomycin D chase assay support a possible post‐transcriptional link between YTHDF3 and CFTR mRNA, but they do not establish m6A‐dependent regulation. AMPK‐related signalling changes were also observed, but the role of this pathway in the YTHDF3‐CFTR‐associated response remains to be clarified. Further studies are needed to determine whether CFTR is directly regulated by YTHDF3 through an m6A‐dependent mechanism and to define how AMPK signalling fits into this process.

## Author Contributions

**Baoxin Tang:** data curation, investigation, writing – review and editing, formal analysis, resources. **Mingkui Gao:** investigation, writing – review and editing, formal analysis, data curation, resources. **Tieyan Li:** conceptualization, methodology, writing – original draft, writing – review and editing. **Chenying Zhu:** investigation, writing – review and editing, formal analysis, data curation, resources. **Heqing Wang:** investigation, writing – review and editing, formal analysis, data curation, resources.

## Funding

This work was supported by the Academic Leaders Training Program of Pudong Health Bureau of Shanghai (Grant No. PWRd2024‐14) awarded to Tieyan Li.

## Disclosure

Artificial Intelligence Statements: While an AI‐powered spell‐checker was used as a final polish, all research, analysis and prose were completed without AI assistance.

## Ethics Statement

All animal protocols were approved by the Ethical Committee of Guangzhou Forevergen Medical Laboratory Animal Center (approval number: IACUC‐AEWC‐F250912001, dated September 12, 2025). Animal experiments were performed in accordance with ARRIVE guidelines and the National Institutes of Health guide for the care and use of Laboratory animals (NIH Publications No. 8023, revised 1978).

## Consent

The authors have nothing to report.

## Conflicts of Interest

The authors declare no conflicts of interest.

## Supporting information


**Figure S1:** Effect of YTHDF3 knockdown on cell function in the OGD/OGR model. (A, B) YTHDF3 expression was confirmed by RT‐qPCR and Western blot after si‐YTHDF3 transfection. AC16 cells were conducted with si‐YTHDF3 transfection and OGD/OGR treatment. C. Cell viability was determined by CCK‐8. D. Cell proliferation was assessed by Edu staining. E. Cell apoptosis was measured by flow cytometry. *N* = 3 independent biological replicates. *p < 0.05, **p < 0.01, ***p < 0.001, ****p < 0.0001.

**Figure S2:** Effects of YTHDF3 and CFTR knockdown on cell viability and apoptosis in the OGD/OGR model. AC16 cells were transfected with si‐YTHDF3 and si‐CFTR, then subjected to OGD/OGR treatment. A. Cell viability was assessed by CCK‐8 assay. B. Edu staining for cell proliferation. C. Flow cytometry for cell apoptosis. *N* = 3 independent biological replicates. *p < 0.05, **p < 0.01, ***p < 0.001, ****p < 0.0001.

**Table S1:** Sequences of target‐specific siRNAs used in this study.

## Data Availability

The original contributions presented in the study are included in the article; further inquiries can be directed to the corresponding author.
