# Supplementary material for: Potential Relationship Between YTHDF3 and CFTR in Myocardial Ischemia–Reperfusion Injury
Source: J Cell Mol Med. 2026 Jun 9;30(11):e71240. doi: 10.1111/jcmm.71240 (PMC13249798; doi:10.1111/jcmm.71240)
Supplement: Supplementary file 3 — Table S1: Sequences of target‐specific siRNAs used in this study. [file JCMM-30-e71240-s002.docx]

**Supplementary Table S1**. Sequences of target-specific siRNAs used in this study

| **siRNA Name** | **Sense strand (5′–3′)** | **Antisense strand (5′–3′)** |
| --- | --- | --- |
| siCFTR-1 | GCAGGUUCUCAGUAGACGA | UCGUCUACUGAGAACCUGC |
| siCFTR-2 | CAUUGUGAUUGGAGCUAUA | UAUAGCUCCAAUCACAAUG |
| siCFTR-3 | GCUCCUAUGUUGUGAUCAU | AUGAUCACAACAUAGGAGC |
| siYTHDF3-1 | GCCUAGUUACUAUGCUCCATT | UGGAGCAUAGUAACUAGGCTT |
| siYTHDF3-2 | GGAUCUCAGGGACAAUCAATT | UUGAUUGUCCCUGAGAUCCTT |
| siYTHDF3-3 | CAUACAUCGUUCCAUUAAATT | UUUAAUGGAACGAUGUAUGTT |
